# Iron and manganese co-limit growth of the Southern Ocean diatom *Chaetoceros debilis*

**DOI:** 10.1371/journal.pone.0221959

**Published:** 2019-09-16

**Authors:** Franziska Pausch, Kai Bischof, Scarlett Trimborn

**Affiliations:** 1 EcoTrace, Alfred Wegener Institute, Helmholtz Centre for Polar and Marine Research, Bremerhaven, Germany; 2 Marine Botany, University of Bremen, Bremen, Germany; University of Nantes, FRANCE

## Abstract

In some parts of the Southern Ocean (SO), even though low surface concentrations of iron (Fe) and manganese (Mn) indicate FeMn co-limitation, we still lack an understanding on how Mn and Fe availability influences SO phytoplankton ecophysiology. Therefore, this study investigated the effects of Fe and Mn limitation alone as well as their combination on growth, photophysiology and particulate organic carbon production of the bloom-forming Antarctic diatom *Chaetoceros debilis*. Our results clearly show that growth, photochemical efficiency and carbon production of *C*. *debilis* were co-limited by Fe and Mn as highest values were only reached when both nutrients were provided. Even though Mn-deficient cells had higher photochemical efficiencies than Fe-limited ones, they, however, displayed similar low growth and POC production rates, indicating that Mn limitation alone drastically impeded the cell’s performance. These results demonstrate that similar to low Fe concentrations, low Mn availability inhibits growth and carbon production of *C*. *debilis*. As a result from different species-specific trace metal requirements, SO phytoplankton species distribution and productivity may therefore not solely depend on the input of Fe alone, but also critically on Mn acting together as important drivers of SO phytoplankton ecology and biogeochemistry.

## Introduction

Large parts of the Southern Ocean (SO) are classified as high-nutrient, low-chlorophyll regions due to the observed low phytoplankton productivity despite high concentrations of macronutrients. The low biomass in these areas results from the very low concentrations of the trace metal iron (Fe) [1], which is required for the optimal growth and cellular function of phytoplankton [2–6]. Fe is integrated in photosystem I and II (PSI and II) and is used in redox reactions in many pathways of the cell, including the electron transport chains of photosynthesis and respiration [4,6,7]. Much less is known whether manganese (Mn) limits or co-limits phytoplankton growth alongside with Fe in the SO [5]. Fe and Mn enter the SO from several sources including atmospheric dust [8,9], upwelling [10], sediments [11,12] and melting of glaciers [13] and sea ice[14]. The flux of mineral-aerosol Fe and Mn into SO waters is probably lower than anywhere else on Earth [1,15]. In fact, concentrations of dissolved Mn in waters of the Drake Passage [16,17] and the Weddell Sea [18] were found to be as low as dissolved Fe concentrations, being generally below 0.4 nmol L^-1^ and potentially (co-)limiting with Fe. Indeed, growth and species composition of phytoplankton assemblages from the Weddell-Scotia Confluence were found to be influenced by enrichment with Fe or Mn alone [19]. Following ash additions to natural phytoplankton assemblages of the Drake Passage, a more significant stimulation of photosynthetic activity and biomass was observed relative to those amended only with Fe [17]. The ash released significantly more Mn than Fe, therefore the strong phytoplankton growth response to ash potentially resulted from the relief of manganese (co-)limitation. According to the three different definitions of co-limitation by [20], the study by [17] hints towards a Type I co-limitation, where two different nutrients do not have the same biochemical function, but are both required for optimal growth. Hence, based on results of the ash-addition experiments Mn can act as a limiting nutrient with Fe in certain parts of the SO. The spatial patterns of Fe-Mn co-limitation across the SO, however, remain largely untested [5].

Manganese is the second most abundant trace metal in thylakoids after Fe [3]. It is essential for phytoplankton growth [21] as it is required in the water-splitting complex of PSII where four Mn ions are involved in the oxidation of water [2]. It is further needed for the antioxidant enzyme superoxide dismutase (SOD), which detoxifies reactive oxygen species (ROS) and prevents cell damage [6,22–24]. To date, the influence of Mn-deficiency on phytoplankton physiology was investigated only in the temperate diatoms *Thalassiosira pseudonana*, *Thalassiosira weissflogii* and *Thalassiosira oceanica* [22,25,26]. These studies revealed that low concentrations of either Fe or Mn alone reduced growth of *T*. *pseudonana* and *T*. *oceanica* and that only with the addition of both metals together highest growth rates were reached, hence demonstrating that both diatoms were co-limited by Fe and Mn [22]. Considering the lack of knowledge on the effects of Fe-Mn co-limitation in particular on Antarctic phytoplankton physiology, the purpose of this study was to elucidate whether Fe and Mn can co-limit the ecologically relevant Antarctic diatom *Chaetoceros debilis*. The latter forms large phytoplankton blooms during spring time [27] and therefore contributes strongly to carbon export in the SO [28]. The strain used for this study was isolated during the European iron fertilization experiment (EIFEX) where it was one of the two species with a continuous exponential growth stimulated by Fe fertilization [29]. The same species also dominated the phytoplankton community after the Fe fertilization experiment SEEDS in the North East Pacific [30]. In this study, the responses in growth rate, photophysiology, pigment composition and particulate organic carbon production of *C*. *debilis* were assessed under altered Fe and Mn availability.

## Material and methods

### Culture conditions

Fe-Mn manipulation experiments were performed with the Antarctic diatom *Chaetoceros debilis* (Polarstern expedition ‘EIFEX’ ANT-XXI/3, In-Patch, 2004, 49° 36 S, 02° 05 E, isolated by Philipp Assmy) which was grown in stock cultures with Fe- and Mn-enriched natural Antarctic seawater (F/2_R_ medium [31]). For the pre-acclimation phase and the main experiment, dilute batch cultures of *C*. *debilis* were transferred into natural FeMn-poor Antarctic seawater (sampled during Polarstern expedition ANT29-2 on September, 20, 2013, 60° 32' S 26° 29' W, Table 1). This seawater was sterile filtered through acid-cleaned filter cartridges (0.2 μm, Sartobran, Sartorius) and spiked with chelexed (Chelex 100, Sigma Aldrich, Merck) macronutrients (100 μmol L^-1^ Si, 100 μmol L^-1^ NO_3_^-^, and 6.25 μmol L^-1^ PO_4_^3-^) and vitamins (30 nmol L^-1^ B_1_, 23 nmol L^-1^ B_7_, and 0.228 nmol L^-1^ B_12_) according to F/2_R_ medium [31]. To represent trace metal concentrations typical for Antarctic high nutrient low chlorophyll waters, a mixture of zinc (0.16 nmol L^-1^), copper (0.08 nmol L^-1^), cobalt (0.09 nmol L^-1^ Co), molybdenum (0.05 nmol L^-1^) was added and adjusted to maintain the ratio of the original F/2 recipe. Our experimental treatments consisted of four different combinations of Fe and Mn concentrations of the culture medium (Table 1) without any Fe or Mn addition (-FeMn treatment) and to which enrichments of Fe alone (-Mn treatment), Mn alone (-Fe treatment) or both trace metals together (control treatment) were made. The trace metals Fe and Mn were added as FeCl_3_ (4 nmol L^-1^, AAS standard, TraceCERT, Fluka) and MnCl_2_ (2 nmol L^-1^, AAS standard, TraceCERT, Fluka). As suggested in [32], in an effort to minimize the alteration of the natural seawater trace metal chemistry and ligands, no ethylenediaminetetraacetic acid (EDTA) was added. Due to the naturally present ligands (1.62 ± 0.20 nmol L^-1^), it is expected that a part of the added Fe was buffered rather than forming inorganic colloids. In order to prevent potential contamination, the experiments were conducted under trace metal clean conditions and therefore all sampling and handling of the incubations was conducted under a laminar flow hood (Class 100, Opta, Bensheim, Germany). Prior to use, the 2 L polycarbonate incubation bottles and other equipment were cleaned for 7 days in a detergent bath containing 1% Citranox solution (Sigma-Aldrich, St. Louis, MO, USA) followed by rinsing 7 times with Milli-Q (Millipore, Merck, Darmstadt, Germany). All bottles and other equipment were subsequently filled with 1 M hydrochloric acid (HCl) for 7 days. After 7 rinsing steps with Milli-Q, all equipment was dried under the clean bench and stored triple-bagged in polyethylene bags until usage.

**Table 1:** Total dissolved Fe (dFe) and Mn (dMn) concentrations from the natural Antarctic seawater as well as from the four different treatments (-FeMn, -Fe, -Mn, control) were determined in the culture medium (0.2 μm filtered Antarctic seawater). Values represent the range of duplicate measurements of the different culture mediums.

	dFe (nmol L^-1^)	dMn (nmol L^-1^)
Seawater	0.30–0.29	0.46–0.52
*Culture medium*:		
-FeMn	0.46–0.48	0.51–0.58
-Fe	0.78–0.83	2.17–2.35
-Mn	3.19–3.70	0.57–0.58
Control	2.52–2.59	2.15–2.17

All incubations were grown at 2°C under a light:dark cycle of 16:8 h at a light intensity of 100 μmol photons m^−2^ s^−1^ using light-emitting diodes (LED) lamps (SolarStinger LED Sun Strip Marine Daylight, Econlux, Cologne, Germany). Light intensities were adjusted using with a light sensor (ULM-500 Universal Light Meter equipped with a Spherical Micro Quantum Sensor US-SQS, Walz GmbH, Effeltrich, Germany). As differences in the maximum quantum yield of photochemistry in PSII (*F*_*v*_/*F*_*m*_) were observed between treatments during pre-acclimation after 15 days, the main experiment was started. Main experiments were conducted in triplicates with a starting cell density of ~ 250 cells mL^-1^ and were run in parallel. During the experiments, the growth rates in all incubations were monitored, confirming exponential growth. Depending on the experimental treatment, cells were harvested after 14 and 18 days with cell densities of 79 042 ± 6 904 cells mL^-1^ in the -FeMn treatment, 93 603 ± 10 805 cells mL^-1^ in the -Mn treatment, 72 155 ± 4158 cells mL^-1^ in the -Fe treatment and 114 819 ± 26 661 cells mL^-1^ in the control treatment. Samples for each treatment were taken at the same time of day to allow a direct comparison of all measured parameters.

### Determination of total dissolved Fe and Mn concentrations of the seawater

Samples to determine total dissolved Fe (dFe) and Mn (dMn) concentrations of the Antarctic seawater and the different culture mediums were taken (Table 1). To this end, 100 mL of each sample were filtered through HCl-cleaned polycarbonate filters (0.2 μm pore size, 47-mm, Nuclepore, Whatman, GE Healthcare, Chicago, IL, USA) using a trace metal clean filtration system under a clean bench. The filtrate was then filled into a 125 mL PE bottle and stored triple-bagged at 2°C until analysis. Between each filtration, the filtration system was cleaned in an acid bath with 1 M HCl and rinsed with Milli-Q.

Concentrations of total dFe and dMn of each seawater sample and process blanks were analysed using a SeaFast system (Elemental Scientific, Omaha, NE, USA) [33,34] coupled to an inductively coupled plasma mass spectrometer (ICP-MS, Element2, Thermo Fisher Scientific, resolution of R = 4000). An iminodiacetate (IDA) chelation column (part number CF-N-0200, Elemental Scientific) was used in the pre-concentration step. All labware used for analysis was pre-cleaned according to the Geotraces cookbook [35]. Prior to the analysis of dFe and dMn, 0.2 μm pre-filtered seawater samples were acidified to pH 1.7 with double distilled HNO and UV-oxidized using a 450 W photochemical UV power supply (ACE GLASS Inc., Vineland N.J., USA). Two blanks were processed the same way during each UV digestion step. The ICP-MS was optimized daily to achieve oxide forming rates below 0.3%. Each seawater sample was analyzed in duplicates via standard addition to minimize any matrix effects, which might influence the quality of the analysis. A pH of 1.7 was needed in order to minimize the formation of Mn and Fe hydroxides and was sufficiently high to minimize the loss of other trace metals on the SeaFast column (pers. comm. Mr. Klemens, Elemental Scientific). To assess the accuracy and precision of the method, a NASS-6 (National Research Council of Canada) reference standard was analyzed in a 1:10 dilution (corresponding to environmentally representative concentrations) at the beginning, during and at the end of a run (two batch runs; n = 6), yielding 487 ± 23 ng L^-1^ (certified 495 ± 46 ng L^-1^) and 546 ± 48 ng L^-1^ (certified 530 ± 50 ng L^-1^) for dFe and dMn, respectively.

### Chlorophyll *a* fluorescence

At the end of the experiments, chlorophyll *a* fluorescence measurements were conducted for each replicate at 2°C using a Fast Repetition Rate fluorometer (FRRf, FastOcean PTX sensor, Chelsea Technologies Group (CTG) Ltd, West Molesey, UK) connected with a FastAct Laboratory system (CTG Ltd). Chlorophyll *a* fluorescence measurements were conducted with cell densities of 79 042 ± 6 904 cells mL^-1^ in the -FeMn treatment, 93 603 ± 10 805 cells mL^-1^ in the -Mn treatment, 72 155 ± 4158 cells mL^-1^ in the -Fe treatment and 114 819 ± 26 661 cells mL^-1^ in the control treatment. Excitation wavelengths of the fluorometer’s LEDs were 450 nm, 530 nm and 624 nm with an automated adjustment of the light intensity (between 0.66–1.2 x 10^22^). The single turnover mode was set with a saturation phase consisting of 100 flashlets on a 2 μs pitch followed by a relaxing phase of 40 flashlets on a 50 μs pitch. After 10 min of dark acclimation, the minimum (*F*_0_) and maximum (*F*_*m*_) chlorophyll *a* fluorescence of PSII was determined 6 times to calculate the maximum quantum yield of photochemistry in PSII (*F*_*v*_/*F*_*m*_, rel. unit) using the equation (Eq.):

$$F_{v}/F_{m}{}_{}=\left(F_{m}‐F_{0}\right)/F_{m}$$ Eq 1

From the single turnover measurements of dark-acclimated cells, also the functional absorption cross section of PSII (σ_PSII,_ nm^2^ PSII^-1^), the time constant for electron transport at the acceptor side of PSII (τ_Qa_, μs) and the connectivity factor (*p*, dimensionless) were derived according to [36], using FastPro8 Software (Version 1.0.55, Kevin Oxborough, CTG Ltd.).

Electron transport rates (ETR)-irradiance curves with sequential increasing irradiances were performed, with 9 irradiances ranging from 0 to 1,700 μmol photons m^-2^ s^-1^ with an acclimation phase of 5 min per light level. Each actual light intensity (E, μmol photons m^-2^ s^-1^) emitted from the FastAct Laboratory system (CTG Ltd.) was measured with a light sensor (ULM-500 Universal Light Meter equipped with a Spherical Micro Quantum Sensor US-SQS, Walz GmbH, Effeltrich, Germany) and used to calculate absolute electron transport rates (ETR, e^-^ PSII^-1^ s^-1^) according to the formula by [37,38]:

$$ETR=\sigma_{PSII}\;x\;\left(\left(F_{q}\prime /F_{m}\prime \right)/\left(F_{v}/F_{m}\right)\right)\;x\;E$$ Eq 2

where *F*_*q*_′/*F*_*m*_′ denotes the effective PSII quantum yield under ambient light. According to [39], maximum ETR (ETR_max_, e^-^ PSII^-1^ s^-1^), light utilization efficiency (*α*) and minimum saturating irradiance (I_k_, μmol photons m^-2^ s^-1^) were calculated from the fitted irradiance-dependent ETR using the SigmaPlot 13.0 software (SysStat Software Inc.).

### Growth

Cell count samples of *C*. *debilis* for each treatment were taken on a daily basis at the same time of the day. All samples were fixed with 10% acid Lugol’s solution and stored at 2°C in the dark until enumeration. At least 400 *C*. *debilis* cells were counted in stripes under a magnification of 400x in combination with a 1.6x optovar using Utermöhl chambers (Hydrobios, Altenholz, Germany) on an inverted microscope (Axio Observer D1, Carl Zeiss AG, Oberkochen, Germany). Cell-specific growth rate (μ) was calculated per day (d^-1^) as

$$\mu =\left(lnN_{t2}–lnN_{t1}\right)/\Delta t$$ Eq 3

where *N*_t1_ and *N*_*t2*_ represent the cell densities (cells mL^-1^) at the sampling day t_1_ and t_2_, respectively, and *Δt* denotes the time between the two measurements.

### Elemental composition

For the analyses of the content of particulate organic carbon (POC) and particulate organic nitrogen (PON), 250 mL of the *C*. *debilis* cultures was gently filtered (< 20 mmHg) onto pre-combusted glass-fibre filters (15h, 500°C, GF/F, ~0.6 μm, 25 mm, Whatman, Wisconsin, USA). Filters were stored in pre-combusted glass petri dishes at -20°C until sample preparation. Prior the analysis, filters were dried at 50°C for > 12 h before they were acidified with 200 μL of 0.2 M HCl to remove inorganic carbon. After being dried at 50°C overnight, filters were coated in tin foil and compressed into small pellets and analysed on an automated carbon nitrogen elemental analyser (Euro EA—CN Elemental Analyzer, HEKAtech GmbH, Wegberg, Germany). Cellular contents of POC and PON were corrected for blank measurements and normalized to cell density and filtered volume. To calculate cellular daily production rates of POC and PON, cellular quotas were multiplied by the corresponding growth rate of the respective treatment. Molar ratios of carbon to nitrogen (C:N) were also calculated.

### Pigment analysis

For the analysis of the photosynthetic pigments, 250 mL of the *C*. *debilis* cultures were gently filtered (< 20 mmHg) onto 25-mm GF/F filters (~0.6 μm, 25 mm, Whatman, Wisconsin, USA), which were then immediately frozen in liquid nitrogen (N_2_) and stored at -80°C until analysis. Pigments samples were homogenized and extracted in 90% acetone for 24 h at 4°C in the dark. After centrifugation (5 min, 4°C, 13000 rpm) and filtration through a 0.45 μm pore size nylon syringe filter (Nalgene^®^, Nalge Nunc International, Rochester, NY, USA), concentrations of chlorophyll *a* and c_2_, fucoxanthin, diatoxanthin and diadinoxanthin were determined by reversed phase high performance liquid chromatography. The analysis was performed on a LaChromElite^®^ system equipped with a chilled autosampler L-2200 and a DAD detector L-2450 (VWR-Hitachi International GmbH, Darmstadt, Germany). A Spherisorb^®^ ODS-2 column (25 cm x 4.6 mm, 5 μm particle size; Waters, Milford, MA, USA) with a LiChropher^®^ 100-RP-18 guard cartridge was used for the separation of pigments, applying a gradient according to [40]. Peaks were detected at 440 nm, identified and quantified by co-chromatography with standards for chlorophyll *a* and c_2_, fucoxanthin, diatoxanthin and diadinoxanthin (DHI Lab Products, Hørsholm, Denmark) using the software EZChrom Elite ver. 3.1.3. (Agilent Technologies, Santa Clara, CA, USA). Pigment contents were normalized to filtered volume and cell densities to yield cellular quotas.

### Statistics

Kolmogorov-Smirnov tests with Liliefors correction were applied to test for normal distribution of the data and Brown-Forsythe tests were applied to test for equal variances. To test for significant differences between treatments one-way analyses of variance (ANOVA) with additional Bonferroni’s multiple comparison post tests were applied using the program GraphPad Prism (Version 5.00 for Windows, Graph Pad Software, San Diego California, USA). The significance testing was done at the *p* < 0.05 level.

## Results

### Total dissolved Fe (dFe) and Mn (dMn) concentrations

The naturally FeMn-poor Antarctic seawater, used for the experiment, contained 0.30 ± 0.01 nmol dFe L^-1^ and 0.49 ± 0.04 nmol dMn L^-1^ (Table 1). Culture media was prepared using this seawater, to which either no Fe and Mn (-FeMn), only Mn (-Fe) or Fe (-Mn) alone or both trace metals together (control) were added. Hence, concentrations of dFe and dMn of the culture medium were altered depending on the treatment (Table 1). While dFe concentrations of the culture medium of the -FeMn and the -Fe treatments were similar, they were reduced relative to ones of the -Mn and control treatments. The culture medium of the -FeMn and -Mn treatments exhibited similar dMn concentrations. In comparison to the latter treatments, dMn concentrations of the culture medium of the -Fe and control treatments were enhanced after Mn enrichment.

### Maximum quantum yield and functional absorption cross-sections of PSII

**Fig 1:**
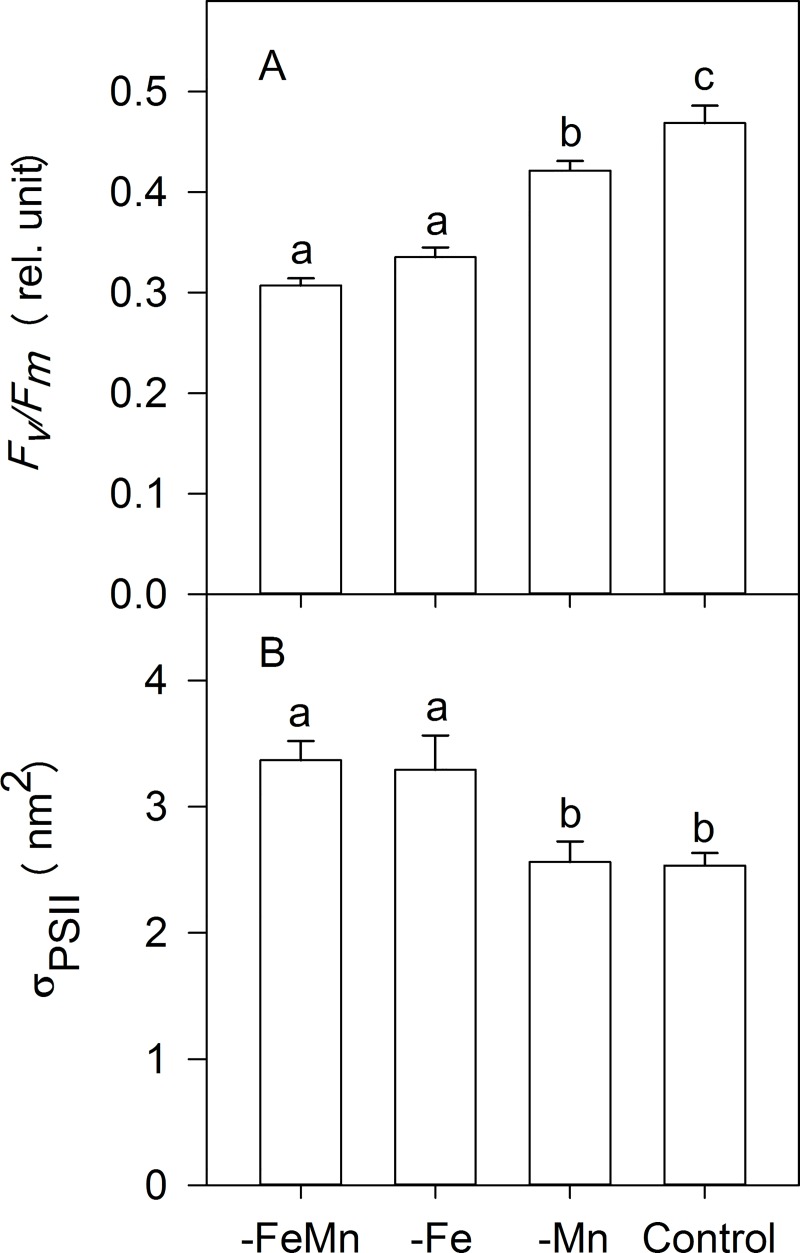
The dark-acclimated maximum PSII quantum yield *F*_*v*_*/F*_*m*_ (A) and the dark-adapted functional absorption cross section of PSII photochemistry σ_PSII_ (B) of *C*. *debilis* grown in naturally FeMn-poor Antarctic seawater (-FeMn) and to which additions of either Mn alone (-Fe), Fe alone (-Mn) or both (control) were given. Values represent the means ± SD (n = 3). Different letters indicate significant differences between treatments (*p* < 0.05).

The maximum quantum yield of PSII (*F*_*v*_*/F*_*m*_) of *C*. *debilis* was significantly influenced by the availability of both trace metals (ANOVA: F = 92, *p* < 0.0001, Fig 1A). While Mn enrichment (-Fe: 0.33 ± 0.01) did not alter the *F*_*v*_*/F*_*m*_ in comparison with cells grown in FeMn-poor water (-FeMn: 0.31 ± 0.01), Fe addition increased the yield, reaching a value of 0.42 ± 0.01. Only after enrichment with both trace metals the *F*_*v*_*/F*_*m*_ of *C*. *debilis* was highest (control: 0.47 ± 0.02).

The functional absorption cross section of PSII (σ_PSII_) was significantly affected by the availability of both trace metals (ANOVA: F = 18.33, *p* = 0.0006, Fig 1B). As for *F*_*v*_/*F*_m_, the addition of Mn alone (-Fe: 3.29 ± 0.27 nm^2^) did not change σ_PSII_ relative to the -FeMn treatment (3.37 ± 0.15 nm^2^). Only when Fe alone (-Mn: 2.56 ± 0.16 nm^2^) or Fe and Mn together (control: 2.53 ± 0.10 nm^2^) were given, σ_PSII_ decreased significantly by 24% and 25%, respectively, relative to the -FeMn treatment. Irrespective of whether Fe alone or both trace metals were added σ_PSII_ remained unchanged.

### Growth, elemental composition and stoichiometry

**Fig 2:**
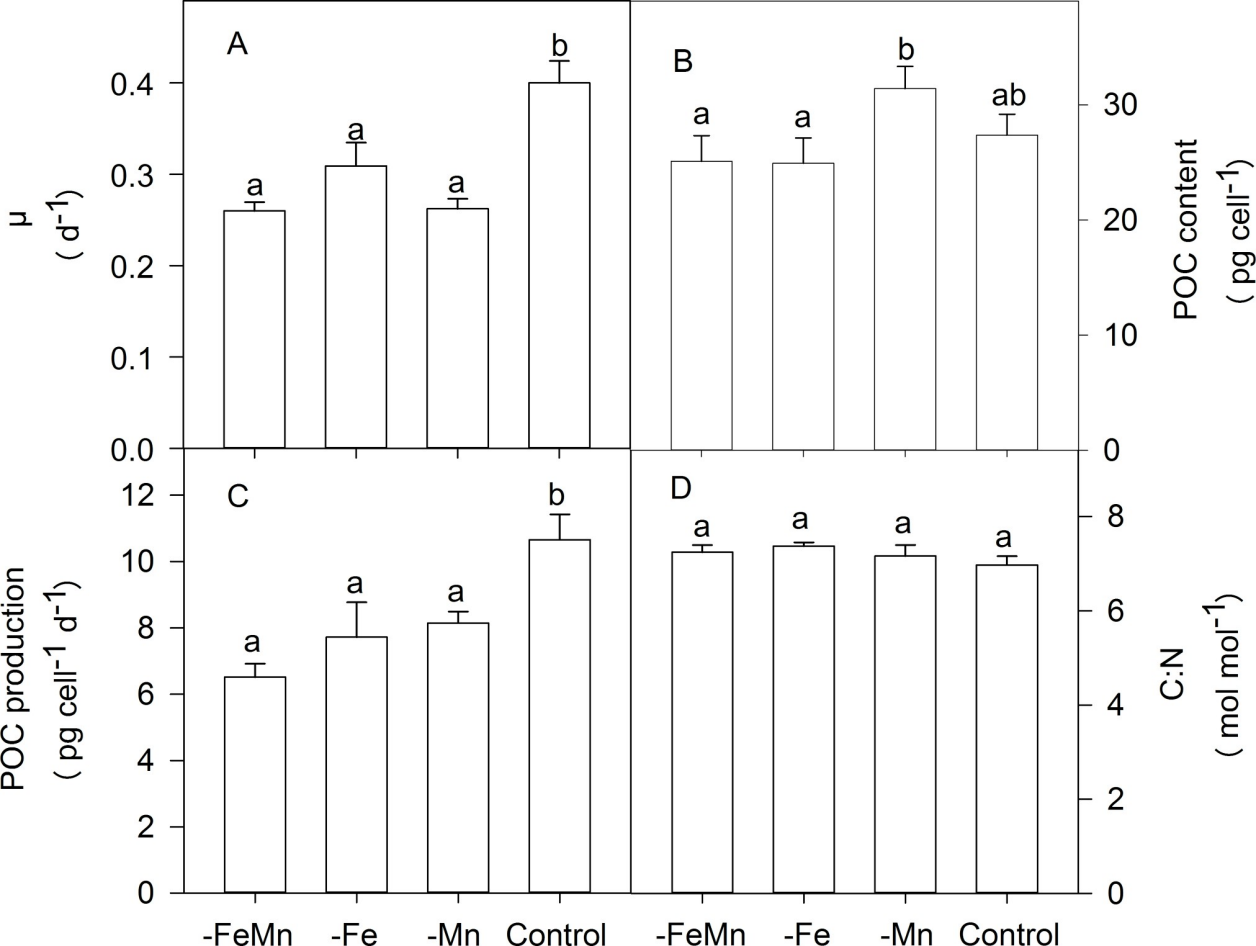
Growth rates (A), cellular production rate of particulate organic carbon (POC, B) and molar ratios of carbon to nitrogen (C:N, C) of *C*. *debilis* grown in naturally FeMn-poor Antarctic seawater (-FeMn) and to which additions of either Mn alone (-Fe), Fe alone (-Mn) or both (control) were given. Values represent the means ± SD (n = 3). Different letters indicate significant differences between treatments (*p* < 0.05).

Growth rates of *C*. *debilis* were significantly altered in response to the different Fe and Mn concentrations (ANOVA: F = 35.67, *p* < 0.0001, Fig 2A). While growth rates were similar between -FeMn (0.26 ± 0.01 d^-1^), -Fe (0.31 ± 0.03 d^-1^) and -Mn (0.26 ± 0.01 d^-1^) treatments, the addition of Fe and Mn together yielded the highest growth rates (0.40 ± 0.02 d^-1^).

Cellular contents of particulate organic carbon (POC) were significantly influenced by different Fe and Mn concentrations (ANOVA: F = 6.508, *p* = 0.0154, Fig 2B). POC quotas were similar for -FeMn (25.11 ± 2.21 pg cell^-1^) and -Fe (24.91 ± 2.21 pg cell^-1^) treatments, they were, however, lower than compared to the -Mn treatment (31.40 ± 1.93 pg cell^-1^). POC quotas of the control treatment (27.38 ± 1.81 pg cell^-1^) were similarly high as all other treatments. POC production rates were significantly affected by Fe and Mn availability (ANOVA: F = 18.38, *p* = 0.0006, Fig 2C). The same trend as for growth was observed for the POC production rates, with the -FeMn (6.51 ± 0.40 pg cell^-1^ d^-1^), -Fe (7.72 ± 1.05 pg cell^-1^ d^-1^) and -Mn (8.14 ± 0.35 pg cell^-1^ d^-1^) treatments showing similar high rates. Again, only when Fe and Mn were added together did POC production reached the highest values of 10.66 ± 0.77 pg cell^-1^ d^-1^. Irrespective of changes in Fe and Mn availabilities, carbon to nitrogen ratios (C:N) remained unchanged among all treatments and ranged between 6.97 ± 0.18 and 7.37 ± 0.08 mol mol^-1^ (Fig 2D).

### Chlorophyll *a* fluorescence

The connectivity of adjacent PSIIs (*p*) was significantly affected in response to the different Fe and Mn concentrations (ANOVA: F = 62.29, *p* < 0.0001, Table 2). Similar *p* values were observed for -FeMn and -Fe treatments. These values were, however, lower compared to the -Mn and control treatments. Between -Mn and control treatments, no difference in *p* was found. The time constant for electron transfer at PSII (τ_Qa_) was significantly altered in response to the different Fe and Mn concentrations (ANOVA: F = 11.38, *p* = 0.0029, Table 2). While τ_Qa_ values did not change when *C*. *debilis* was grown under -FeMn or -Fe conditions, these values were, however, significantly higher when Fe was added alone (-Mn) or in combination with Mn (control). The only exception was the -Fe treatment, for which τ_Qa_ did not differ from the value determined for the -Mn treatment.

**Table 2:** Connectivity between adjacent photosystems (*p*), time constant for electron transfer at PSII (τ_Qa_), absolute maximum electron transport rates (ETR_max_), light saturation point (I_k_) and light use efficiency (α) were measured for *C*. *debilis* grown in naturally FeMn-poor Antarctic seawater (-FeMn) and to which additions of either Mn alone (-Fe), Fe alone (-Mn) or both (control) were given. Values represent the means ± SD (n = 3). Different letters indicate significant differences between treatments (*p* < 0.05).

Treatment	*P* (rel. unit)	τ_Qa_ (*μ*s)	ETR_max_ (e^-^ PSII^-1^ s^-1^)	I_k_ (*μ*mol photons m^-2^ s^-1^)	α (rel. unit)
-FeMn	0.32 ± 0.01^a^	426 ± 29^a^	869 ± 150^a^	495 ± 90^a^	1.81 ± 0.08^a^
-Fe	0.35 ± 0.01^a^	461 ± 39^ab^	945 ± 72^a^	532 ± 35^a^	1.78 ± 0.16^a^
-Mn	0.42 ± 0.01^b^	510 ± 27^bc^	531 ± 64^b^	408 ± 26^a^	1.30 ± 0.12^b^
Control	0.45 ± 0.02^b^	551 ± 12^c^	528 ± 50^b^*	427 ± 89^a^*	1.21 ± 0.07^b^*

*Please note that the marked values need to be treated with caution as ETRs values for the two highest irradiances (1152 and 1504 μmol photons m^−2^ s^−1^) of the control treatment could unfortunately not be determined due to a technical problem with the FRRf software FastPro8.

**Fig 3:**
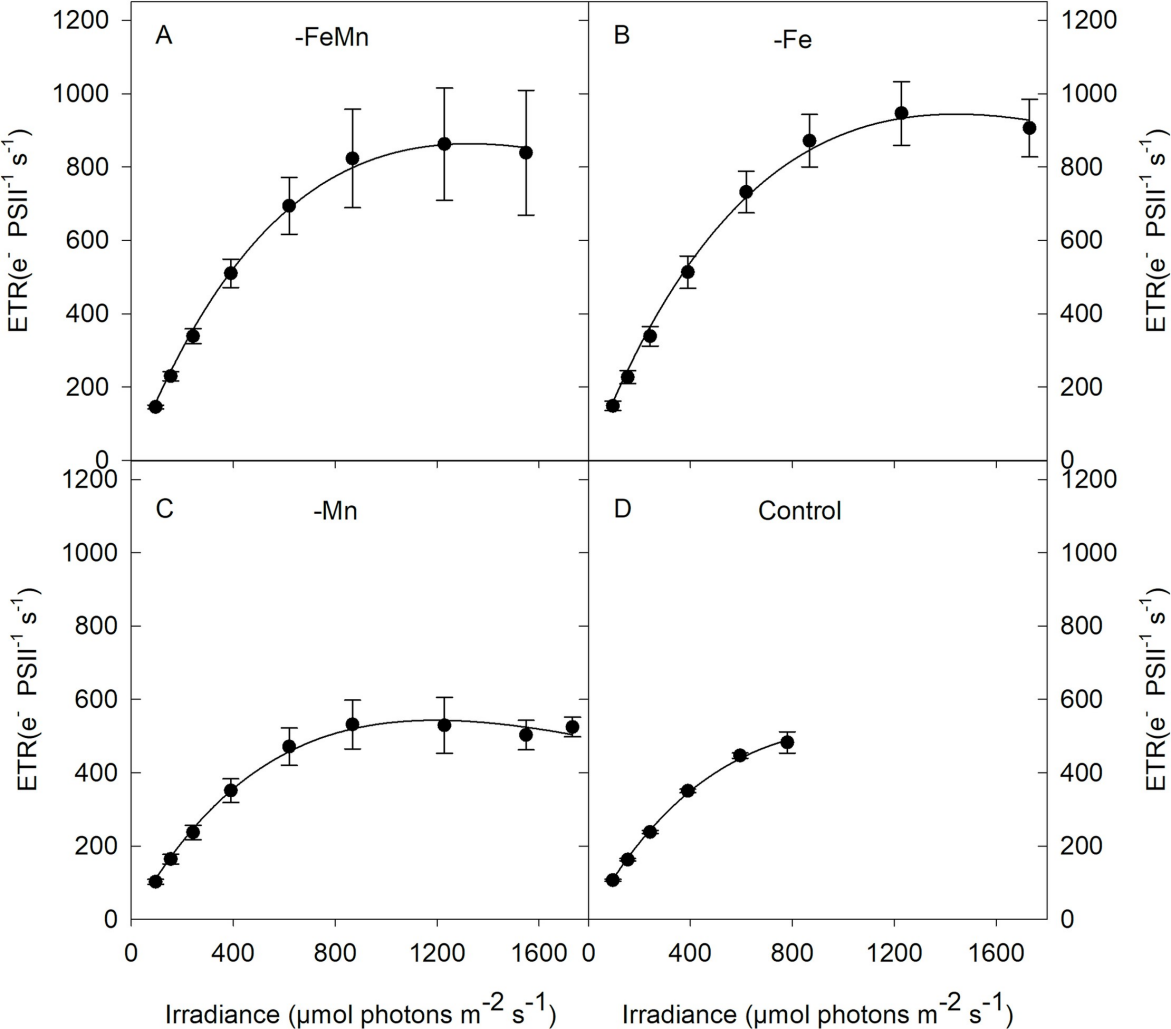
Absolute electron transport rates (ETR) were measured in response to increasing irradiance in *C*. *debilis* grown in naturally FeMn-poor Antarctic seawater (-FeMn, A) and to which additions of either Mn alone (-Fe, B), Fe alone (-Mn, C) or both (control, D) were given. Values represent the means ± SD (n = 3).

ETR-irradiance curves showed differences in both shape and amplitude in response to changes in Fe and Mn availability (Fig 3). Maximum electron transport rates (ETR_max_) were significantly affected by the different trace metal additions (ANOVA: F = 17.63, *p* = 0.0007, Table 2). ETR_max_ values were similar for the -FeMn and -Fe treatments, but were higher than those measured of the -Mn and control treatments. Please note that ETR values for the two highest irradiances (1152 and 1504 μmol photons m^−2^ s^−1^) of the control treatment could unfortunately not be determined due to a technical problem with the FRRf software FastPro8. For unknown reasons the software did not follow the protocol during the measurements for these two light intensities as no dark acclimation of 5 minutes was performed. Due to this, these data were excluded from the analysis. Calculating ETR_max_ using the light-adapted σ_PSII_^’^ showed the same trend. The light saturation point of PSII electron transport (I_k_) ranged between 408 ± 26 and 532 ± 35 μmol photons m^-2^ s^-1^ among treatments and were neither influenced by the addition of Fe or Mn alone nor the combination of both (Table 2). The light use efficiency (α) was significantly influenced by the availability of Fe and Mn (ANOVA: F = 23.09, *p* = 0.0003, Table 2), with α values of the -FeMn and -Fe treatments being higher than those of the -Mn and control treatments. In comparison, α remained unaltered between the -FeMn and -Fe as well as between the -Mn and control treatments.

### Pigments

Cellular concentrations of light-harvesting pigments (LH = chlorophyll *a* + chlorophyll c_2_ + fucoxanthin) were significantly altered by the availability of Fe and Mn (ANOVA: F = 24.21, *p* = 0.0002, Table 3). LH quotas remained unchanged when *C*. *debilis* was grown under -FeMn or -Fe conditions, these values were, however, increased when Fe was added alone (-Mn) or in combination with Mn (control). Between -Mn and control treatments, there was no difference in LH quotas. The availability of Fe and Mn influenced cellular concentrations of light-protective pigments (LP = diadinoxanthin + diatoxanthin) (ANOVA: F = 7.317, *p* = 0.0111, Table 3). While LP quotas of the -Fe and control treatments differed, LH quotas were similar among the other treatments. The availability of Fe and Mn had a strong influence on LH:LP ratios (ANOVA: F = 7.273, *p* < 0.0133, Table 3). Only after enrichment with both trace metals the LH:LP ratio become the smallest relative to all other treatments. In comparison, LH:LP ratios were similar between -FeMn, -Fe and -Mn treatments.

**Table 3:** Cellular concentrations of light-harvesting (LH: sum of chlorophyll *a*, chlorophyll c_2_, and fucoxanthin) and light-protective pigments (LP: sum of diadinoxanthin and diatoxanthin) as well as the ratio of light-protective to light-harvesting pigments (LP:LH) were determined in *C*. *debilis* grown in naturally FeMn-poor Antarctic seawater (-FeMn) and to which additions of either Mn alone (-Fe), Fe alone (-Mn) or both (control) were given. Values represent the means ± SD (n = 3). Different letters indicate significant differences between treatments (*p* < 0.05).

Treatment	LH (fg cell^-1^)	LP (fg cell^-1^)	LP:LH (%)
-FeMn	89 ± 2^a^	58 ± 0^ab^	0.72 ± 0.07^a^
-Fe	80 ± 2^a^	56 ± 6^a^	0.74 ± 0.06^a^
-Mn	118 ± 5^b^	70 ± 4^ab^	0.73 ± 0.08^a^
Control	135 ± 17^b^	71 ± 7^b^	0.56 ± 0.01^b^

## Discussion

Previous studies reported that growth and species composition of phytoplankton assemblages of different SO regions was influenced solely by the addition of Mn [1,17,19]. As these studies were undertaken with natural phytoplankton assemblages, it has not yet been investigated how Mn and Fe availability influences SO phytoplankton physiology and which species are actually susceptible to the limitation of these two trace metals. This study shows that growth of the Antarctic bloom-forming diatom *C*. *debilis* was co-limited by Fe and Mn and that Mn deficiency alone inhibited growth and carbon production to the same degree as Fe limitation. Therefore, we define Fe-Mn co-limitation according to [20] as “Type I: Independent nutrient co-limitation”.

### FeMn- and Fe-deficient cells showed similar physiological responses

The determination of dFe and dMn concentrations in the culture medium of all treatments revealed that the four experimental treatments were successfully achieved (Table 1). In line with this, at the time of sampling the *F*_*v*_/*F*_*m*_ of FeMn-deficient cells (0.31 ± 0.01) was identical with the one of Fe-deficient cells (0.34 ± 0.01), but strongly reduced compared to FeMn-replete cells (0.47 ± 0.02; Fig 1A). Hence, the similar low *F*_*v*_/*F*_*m*_ values of the FeMn- and Fe-deficient cells suggest reduced photochemical efficiency, a common feature of Fe-limited phytoplankton [7,41–44]. To counterbalance the reduced amount of Fe-containing photosynthetic reaction centers [41,43,45,46], both FeMn- and Fe-poor cells displayed larger *σ*_PSII_ relative to the FeMn-replete cells (Fig 1B). A Fe-dependent disconnection of antennae from PSII reaction centres [42,45,47,48] was also apparent for FeMn- and Fe-deficient cells (Table 2). Hence, the photophysiological response of the FeMn-deficient incubations was largely driven by low Fe availability. Along with these photophysiological changes, FeMn- and Fe-deficient cells also displayed significantly lower growth, and POC production rates relative to the FeMn-replete cells (Fig 2A, 2B and 2D). Similar low growth and carbon fixation rates were previously estimated under Fe limitation alone in the same species [49,47], with other *Chaetoceros* species [42,50] and with various other Antarctic diatoms [45,51]. In a previous study by [47], a smaller cell volume of the same *C*. *debilis* strain was observed under Fe-limitation alone. However, POC quotas were reduced as a consequence of Fe-limitation after normalization to either cell number or cell volume. Since the cell size was not measured for this experiment and therefore no cell volume-normalized POC quotas could be derived, we assume based on [47] that not only cell volume decreased under Fe-limitation, but also the cell volume-normalized POC content per cell of *C*. *debilis*.

Low Fe availability usually causes chlorosis [45,51–54], as seen by the reduced light harvesting pigment quotas of both FeMn- and Fe-deplete cells (Table 3). Moreover, Fe is required in various components of the electron transport chain: as a consequence of Fe-limitation, fewer Fe-containing proteins are synthesized, interrupting the transfer of electrons in the photosynthetic electron transport chain [55]. Electron transfer was largely hindered under Fe-limiting conditions [4,42,51]. Hence, POC production rates were significantly lower in both FeMn- and Fe-deplete relative to the FeMn enriched cells (Fig 2C). This generally resulted in reduced rates of photosynthesis [42,45,51,54,56], which affects cellular growth rates (Fig 2A). Unexpectedly, re-oxidation time of Q_a_ was shorter (Table 2) while electron transfer of both FeMn- and Fe-deficient cells was significantly enhanced relative to FeMn-replete cells (Fig 3, Table 2). The higher ETRs combined with reduced carbon fixation suggest that electrons entered alternative pathways like the Mehler reaction [57] and the cyclic electron transport flow around PSI. Considering, however, that the latter pathways are rather Fe-expensive, less Fe-expensive processes such as the putative plastid plastoquinol terminal oxidase (PTOX) pathway were of importance [58]. Furthermore, minimum saturating irradiance (I_k_) as well as light utilization efficiency (α) were both enhanced in the FeMn- and Fe-deplete relative to the control treatment, indicating enhanced acclimation to short-term high light stress [4]. The potential to counteract short-term high light stress potentially resulted from the elevated ratios of LP:LH (Table 3), indicating improved capacity of FeMn- and Fe-deficient cells to cope with high light exposure than the FeMn-enriched cells.

### Mn deficiency inhibited growth of *C*. *debilis*

As the water-splitting complex of PSII contains four Mn ions, a lack of Mn can affect the photochemical activity of the overall pool of PSII as previously observed in temperate diatoms [22]. Accordingly, the photochemical efficiency of Mn-deplete cells was reduced relative to FeMn-sufficient cells, but still much higher than FeMn- and Fe-deficient cells. Apparently, the addition of Fe alone to Mn-deplete cells led to restoration of photophysiological adjustments, with better connected adjacent PSIIs, higher PSII photochemical efficiency as well as smaller PSII functional absorption cross sections (Fig 1A and 1B, Table 2). As a consequence, Mn-deficient cells exhibited efficient linear electron transport, which resulted in enhanced POC build-up relative to the FeMn- and Fe-poor incubations (Figs 2B and 3, Table 2). These findings suggest that amendment of Fe alone led to the reorganization of the thylakoid membrane enabling Mn-deficient cells undisturbed linear electron flow, which provided ATP and NADPH for subsequent carbon fixation. Even though photosynthetic activity was restored, the Mn-deficient cells grew as slowly as the FeMn- and Fe-deplete incubations (Fig 2B). Reduced growth of Mn-limited temperate diatoms was previously reported [22,25] and was explained by their impacted capacity of the antioxidant enzyme superoxide dismutase (SOD). The latter detoxifies reactive oxygen species (ROS) and prevents cell damage, but requires Mn. As a consequence, Mn-deficient cells were probably compromised by reduced activity of the Mn-containing SOD, increasing oxidative stress and the requirements for energy and resources to repair damaged cellular constituents [22]. In support for this, to counteract light stress Mn-deficient cells displayed a higher LP:LH ratio than cells grown under FeMn-replete conditions (Table 3). The distinct response between Mn- and Fe-deficient cells further supports the finding of [22] that Fe and Mn do not substitute one another.

### Only the supply of both Fe and Mn yielded highest growth

Only when Fe and Mn were provided together, was maximum photosynthetic efficiency reached, which allowed unimpeded entry of electrons into the photosynthetic electron transport chain (Fig 1A). Similarly, the additions of Mn-containing volcanic ashes to natural communities from the Drake passage resulted in a stronger increase of the *F*_*v*_/*F*_*m*_ than to those in which only Fe was added [17]. Here, only the addition of both metals allowed *C*. *debilis* to achieve maximum POC production and to reach highest growth rates (Fig 2A and 2C), indicating relief from FeMn co-limitation. Similarly, growth rates reached maximum values only when cultures of the two diatoms *T*. *pseudonana* and *T*. *oceanica* were enriched with both trace metals together [22]. Sufficient quantities of Mn-containing SOD and therefore less oxidative stress [22] were indicated by the low LP:LH ratio of FeMn-replete *C*. *debilis* cells compared to all other treatments (Table 3).

The results of this study hint towards the finding that low phytoplankton growth and productivity in SO open ocean waters may not be explained solely by the limitation of Fe, but also with Mn together. In fact, *C*. *debilis* was found to be co-limited by Fe and Mn at similarly low concentrations as previously reported in the Drake Passage [17,18]. As FeMn- and Fe-deficient *C*. *debilis* cells showed similar physiological responses, we hypothesize that the occurrence of FeMn-limited phytoplankton species may be masked in the natural environment. Fe-Mn-enrichment experiments using different SO phytoplankton communities will be helpful to identify Mn and Fe co-limited species, but also such species that have very low requirements of both metals and therefore are better adapted to thrive in FeMn-poor waters. Because of the potential ecological and biogeochemical implications, a mechanistic understanding of photosynthesis and trace metal requirements of SO phytoplankton is needed. Variability in stoichiometries of trace metal supply and biological demand are key determinants of trace metal limitation and as such for phytoplankton productivity. Deciphering the mechanisms that underpin this variability and the consequences for SO phytoplankton species is crucial for accurately predicting the consequences of ongoing anthropogenic perturbations to SO biogeochemistry.

## References

[1] Martin JH, Gordon RM, Fitzwater SE. Iron in Antarctic waters. Nature. 1990; 345: 156–158.

[2] Raven JA. Predictions of Mn and Fe use efficiencies of phototrophic growth as a function of light availability for growth and of C assimilation pathway. New Phytol. 1990; 116: 1–18.

[3] Raven JA, Evans MCW, Korb RE. The role of trace metals in photosynthetic electron transport in O_2_-evolving organisms. Photosyn. Res. 1999 60; 111–149.

[4] Behrenfeld MJ, Milligan AJ. Photophysiological expressions of iron stress in phytoplankton. Annu Rev Mar Sci. 2013; 5: 217–246. 10.1146/annurev-marine-121211-172356 22881354

[5] Moore CM, Mills MM, Arrigo KR, Berman-Frank I, Bopp L, Boyd PW, et al Processes and patterns of oceanic nutrient limitation. Nature Geoscience. 2013; 6: 701–710.

[6] Twining BS, Baines SB. The trace metal composition of marine phytoplankton. Annu Rev Mar Sci. 2013; 5: 191–215. 10.1146/annurev-marine-121211-172322 22809181

[7] Greene RM, Geider RJ, Kolber Z, Falkowski PG. Iron-induced changes in light harvesting and photochemical energy conversion processes in eukaryotic marine algae. Plant Physiol 1992; 100: 565–575. 10.1104/pp.100.2.565 16653030 PMC1075596

[8] Moore JK, Braucher O. Sedimentary and mineral dust sources of dissolved iron to the world ocean. Biogeosciences. 2008; 5: 631–656.

[9] Boyd PW, Ellwood MJ. The biogeochemical cycle of iron in the ocean. Nat Geosci. Nature Publishing Group; 2010; 3: 675–682. 10.1038/ngeo964

[10] de Baar HJW, de Jong JTM, Bakker DCE, Löscher BM, Veth C, Bathmann U V., et al Importance of iron for plankton blooms and carbon dioxide drawdown in the Southern Ocean. Nature. 1995; 373: 412–415. 10.1038/373412a0

[11] De Jong J, Schoemann V, Lannuzel D, Croot P, De Baar H, Tison JL. Natural iron fertilization of the Atlantic sector of the Southern Ocean by continental shelf sources of the Antarctic Peninsula. J Geophys Res Biogeosciences. 2012; 117 10.1029/2011JG001679

[12] Middag R, de Baar HJW, Klunder MB, Laan P. Fluxes of dissolved aluminum and manganese to the Weddell Sea and indications for manganese co-limitation. Limnol Oceanogr. 2013; 58: 287–300. 10.4319/lo.2013.58.1.0287

[13] Raiswell R, Tranter M, Benning LG, Siegert M, De’ath R, Huybrechts P, et al Contributions from glacially derived sediment to the global iron (oxyhydr)oxide cycle: Implications for iron delivery to the oceans. Geochim Cosmochim Acta. 2006; 70: 2765–2780. 10.1016/j.gca.2005.12.027

[14] Sedwick PN, Ditullio GR. Regulation of algal blooms in Antarctic shelf waters by the release of iron from melting sea ice. Geophys Res Lett. 1997; 24: 2515–2518.

[15] Duce RA, Tindale NW. Atmospheric transport of iron and its deposition in the ocean. Limnol Oceanogr. 1991: 36; 1715–1726.

[16] Middag R, de Baar HJW, Klunder MB, Laan P. Fluxes of dissolved aluminum and manganese to the Weddell Sea and indications for manganese co-limitation. Limnol Oceanogr. 2013; 8: 287–300.

[17] Browning TJ, Bouman HA, Henderson GM, Mather TA, Pyle DM, Schlosser C, et al Strong responses of Southern Ocean phytoplankton communities to volcanic ash. Geophys Res Lett. 2014; 41: 2851–2857.

[18] Middag R, de Baar HJW, Laan P, Cai PH, Van Ooijen DJV. Dissolved manganese in the Atlantic sector of the Southern Ocean. Deep-Sea Res. II. 2011; 58: 2661–2677.

[19] Buma AGJ, de Baar HJW, Nolting RF, Van Bennekom AJ. Metal enrichment experiments in the Weddell-Scotia Seas: Effects of iron and manganese on various plankton communities. Limnol Oceanogr. 1991; 36: 1865–1878.

[20] Saito MA, Goepfert TJ, Ritt JT. Some thoughts on the concept of colimitation: Three definitions and the importance of bioavailability. Limnol Oceanogr. 2008; 53: 276–290. 10.4319/lo.2008.53.1.0276

[21] Morel FMM, Price NM. The biogeochemical cycles of trace metals in the oceans. Science. 2003; 300: 944–947. 10.1126/science.1083545 12738853

[22] Peers G, Price NM. A role for manganese in superoxide dismutases and growth of iron-deficient diatoms. Limnol Oceanogr. 2004; 49: 1774–1783.

[23] Wolfe-Simon F, Grzebyk D, Schofield O, Falkowski PG. The role and evolution of superoxide dismutases in algae. J Phycol. 2005; 41: 453–465.

[24] Allen MD, Kropat J, Tottey S, DelCampo JA, Merchant SS. Manganese deficiency in *Chlamydomonas* results in loss of photosystem II and MnSOD function, sensitivity to peroxides, and secondary phosphorus and iron deficiency. Plant Physiol. 2006; 143: 263–277. 10.1104/pp.106.088609 17085511 PMC1761973

[25] Sunda WG, Huntsman SA. Effect of competitive interactions between manganese and copper on cellular manganese and growth in estuarine and oceanic species of the diatom *Thalassiosira*. Limnol Oceanogr. 1983; 28: 924–934.

[26] Sunda WG, Huntsman SA. Antagonisms between cadmium and zinc toxicity and manganese limitation in a coastal diatom. Limnol Oceanogr. 1996; 41: 373–387.

[27] Thomson PG, McMinn A, Kiessling I, Watson M, Goldsworthy PM. Composition and succession of dinoflagellates and chrysophytes in the upper fast ice of Davis Station, East Antarctica. Polar Biol. 2006; 29: 337–345. 10.1007/s00300-005-0060-y

[28] Smetacek V, Assmy P, Henjes J. The role of grazing in structuring Southern Ocean pelagic ecosystems and biogeochemical cycles. Antarct Sci. 2004; 16: 541–558. 10.1017/S0954102004002317

[29] Assmy P, Henjes J, Klaas C, Smetacek V. Mechanisms determining species dominance in a phytoplankton bloom induced by the iron fertilization experiment EisenEx in the Southern Ocean. Deep Res Part I Oceanogr Res Pap. 2007; 54: 340–362. 10.1016/j.dsr.2006.12.005

[30] Tsuda A, Kiyosawa H, Kuwata A, Mochizuki M, Shiga N, Saito H, et al Responses of diatoms to iron-enrichment (SEEDS) in the western subarctic Pacific, temporal and spatial comparisons. Prog Oceanogr. 2005; 64: 189–205. 10.1016/j.pocean.2005.02.008

[31] Guillard RRL, Ryther JH. Studies of marine planktonic diatoms: I. *Cyclotella nana* Hustedt, and *Detonula confervacea* (Cleve) Gran. Can J Microbiol. 1962; 8: 229–239. 10.1139/m62-029 13902807

[32] Gerringa LJA, de Baar HJW, Timmermans KR. A comparison of iron limitation of phytoplankton in natural oceanic waters and laboratory media conditioned with EDTA. Mar Chem 68, 335–346 (2000).

[33] Hathorne EC, Haley B, Stichel T, Grasse P, Zieringer M, Frank M. Online preconcentration ICP-MS analysis of rare earth elements in seawater. Geochem Geophys Geosyst. 2012; 13(1): Q01020.

[34] Rapp I, Schlosser C, Rusiecka D, Gledhill M, Achterberg EP. Automated preconcentration of Fe, Zn, Cu, Ni, Cd, Pb, Co, and Mn in seawater with analysis using high-resolution sector field inductively-coupled plasma mass spectrometry. Anal Chim Acta. 2017;976: 1–13. 10.1016/j.aca.2017.05.008 28576313

[35] Cutter G, Casciotti K, Croot P, Geibert W, Heimbürger L-E, Lohan M, et al Sampling and Sample-handling Protocols for GEOTRACES Cruises. Version 3, Toulouse, France, GEOTRACES International Project Office; 2017 pp. 139 & Appendices.

[36] Oxborough K, Moore CM, Suggett DJ, Lawson T, Chan HG, Geider RJ. Direct estimation of functional PSII reaction center concentration and PSII electron flux on a volume basis: a new approach to the analysis of Fast Repetition Rate fluorometry (FRRf) data. Limnol Oceanogr Methods. 2012; 10: 142–154.

[37] Suggett DJ, MacIntyre HL, Geider RJ. Evaluation of biophysical and optical determinations of light absorption by photosystem II in phytoplankton. Limnol Oceanogr Methods. 2004; 2: 316–332.

[38] Suggett DJ, Moore CM, Hickman AE, Geider RJ. Interpretation of fast repetition rate (FRR) fluorescence: Signatures of phytoplankton community structure versus physiological state. Mar Ecol Prog Ser. 2009; 376: 1–19.

[39] Ralph PJ, Gademann R. Rapid light curves: A powerful tool to assess photosynthetic activity. Aquatic Botany. 2005; 82: 222–237.

[40] Wright SW, Jeffrey SW, Manoura RFC, Llewellyn CA, Bjornland T, Repeta D, et al Improved HPLC method for the analysis of chlorophylls and carotenoids from marine phytoplankton. Mar Ecol Prog Ser. 1991; 77: 183–196.

[41] Hopkinson BM, Mitchell BG, Reynolds RA, Wang H, Selph KE, Measures I, et al Iron limitation across chlorophyll gradients in the southern Drake Passage: Phytoplankton responses to iron addition and photosynthetic indicators of iron stress. Limnol Oceanogr. 2007; 52: 2540–2554.

[42] Petrou K, Trimborn S, Rost B, Ralph P, Hassler CS. The impact of iron limitation on the physiology of the Antarctic diatom *Chaetoceros simplex*. Mar Biol. 2014; 161: 925–937. 10.1007/s00227-014-2392-z 24719494 PMC3969518

[43] Trimborn S, Hoppe CJM, Taylor BB, Bracher A, Hassler CS. Physiological characteristics of open ocean and coastal phytoplankton communities of Western Antarctic Peninsula and Drake Passage waters. Deep Sea Res. Part I: Oceanogr Res Papers. 2015; 98: 115–124.

[44] Trimborn S, Brenneis T, Hoppe CJM, Laglera LM, Norman L, Santos-Echeandìa J, et al Iron sources alter the response of Southern Ocean phytoplankton to ocean acidification. Mar Ecol Prog Ser. 2017; 578: 35–50.

[45] Strzepek RF, Hunter KA, Frew RD, Harrison PJ, Boyd PW. Iron-light interactions differ in Southern Ocean phytoplankton. Limnol Oceanogr. 2012; 57: 1182–1200.

[46] Schuback N, Schallenberg C, Duckham C, Maldonado MT, Tortell PD. Interacting effects of light and iron availability on the coupling of photosynthetic electron transport and CO_2_ assimilation in marine phytoplankton. PloS One. 2015; 10(7): e0133235 10.1371/journal.pone.0133235 26171963 PMC4501554

[47] Trimborn S, Thoms S, Bischof K, Beszteri S. Susceptibility of two Southern Ocean phytoplankton key species to iron limitation and high light. Front Mar Sci. 2019; 6: 167 10.3389/FMARS.2019.00167

[48] Behrenfeld MJ, Kolber ZS. Widespread Iron Limitation of Phytoplankton in the South Pacific Ocean. Science. 1999; 283: 840–843. 10.1126/science.283.5403.840 9933166

[49] Hoffmann LJ, Peeken I, Lochte K. Iron, silicate, and light co-limitation of three Southern Ocean diatom species. Polar Biol. 2008; 31: 1067–1080.

[50] Timmermans K, Davey M, van der Wagt B, Snoek J, Geider R, Veldhuis M, et al Co-limitation by iron and light of *Chaetoceros brevis*, *C*. *dichaeta* and *C*. *calcitrans* (Bacillariophyceae). Mar Ecol Prog Ser. 2001; 217: 287–297.

[51] Alderkamp A-C, Kulk G, Buma AGJ, Visser RJW, Van Dijken GL, Mills MM, et al The effect of iron limitation on the photophysiology of *Phaeocystis antarctica* (Prymnesiophyceae) and *Fragilariopsis cylindrus* (Bacillariophyceae) under dynamic irradiance. J Phycol. 2012; 48: 45–59. 10.1111/j.1529-8817.2011.01098.x 27009649

[52] Reinbothe C, Bartsch S, Eggink LL, Hoober JK, Brusslan J, Andrade-Paz R, et al A role for chlorophyllide a oxygenase in the regulated import and stabilization of light-harvesting chlorophyll *a/b* proteins. Proc Natl Acad Sci. 2006; 103: 4777–4782. 10.1073/pnas.0511066103 16537436 PMC1450246

[53] Van Leeuwe MA, Stefels J. Effects of iron and light stress on the biochemical composition of Antarctic *Phaeocystis sp*. (Prymnesiophyceae). II. Pigment composition. J Phycol. 1998; 34: 496–503.

[54] Koch F, Beszteri S, Harms L, Trimborn S. The impacts of iron limitation and ocean acidification on the cellular stoichiometry, photophysiology and transcriptome of *Phaeocystis antarctica*. Limnol Oceanogr. 2019; 64: 357–375.

[55] Geider RJ, La Roche J. The role of iron in phytoplankton photosynthesis, and the potential for iron-limitation of primary productivity in the sea. Photosyn Res. 1994; 39: 275–301. 10.1007/BF00014588 24311126

[56] Greene RM, Geider RJ, Falkowski PG. Effect of iron limitation on photosynthesis in a marine diatom. Limnol Oceanogr. 1991; 36: 1772–1782.

[57] Mehler AH. Studies on reactions of illuminated chloroplasts. I. Mechanism of the reduction of oxygen and other Hill reagents. Arch Biochem Biophys. 1957; 33: 65–77. 10.1016/0003-9861(51)90082-3 14857775

[58] Mackey KRM, Paytan A, Grossman AR, Bailey S. A photosynthetic strategy for coping in a high-light, low-nutrient environment. Limnol Oceanogr. 2008; 53: 900–913.

